# Regulation of cell proliferation and cell density by the inorganic phosphate transporter PiT1

**DOI:** 10.1186/1747-1028-7-7

**Published:** 2012-03-06

**Authors:** Kristina Byskov, Nina Jensen, Iben Boutrup Kongsfelt, Maria Wielsøe, Lasse Ebdrup Pedersen, Christa Haldrup, Lene Pedersen

**Affiliations:** 1 Department of Molecular Biology and Genetics, Aarhus University, 8000 Aarhus C, Denmark; 2 Institute of Clinical Medicine, Aarhus University, 8000 Aarhus C, Denmark; 3 Department of Hematology, Aarhus University Hospital, 8000 Aarhus C, Denmark; 4 Department of Molecular Biology and Genetics, Aarhus University, C. F. Møllers Allé 3, Building 1130, DK-8000 Aarhus C, Denmark

**Keywords:** PiT1, SLC20A1, inorganic phosphate transporter, cell proliferation, cell density, transformation

## Abstract

**Abstact:**

**Background**

The inorganic phosphate (P_i_) transporter, PiT1 (SLC20A1), is ubiquitously expressed in mammalian cells. It has previously been shown that down-regulation of PiT1 severely impaired the proliferation of two transformed human cells lines, HepG2 and HeLa, and the tumorigenicity of HeLa cells in nude mice. Moreover, PiT1 knock-out mice do not survive past E12.5 and from E10.5, the embryos were found to be growth-retarded and showed reduced proliferation of liver cells. Isolated mouse embryonic fibroblasts with knocked out as well as reduced PiT1 expression levels also exhibited impaired proliferation. Together these results suggest that a certain level of PiT1 is important for proliferation. We have here investigated the role of PiT1 in regulation of cell proliferation using two strictly density-inhibited cells lines, the murine MC3T3-E1 and NIH3T3 cells.

**Results**

We found that knock-down of PiT1 in MC3T3-E1 cells led to impaired proliferation supporting that at least a certain level of PiT1 is important for wildtype level of proliferation. We, however, also observed that MC3T3-E1 and NIH3T3 cells themselves regulate their endogenous PiT1 mRNA levels with lower levels in general correlating with decreased proliferation/increased cell density. Moreover, over-expression of human PiT1 led to increased proliferation of both MC3T3-E1 and NIH3T3 cultures and resulted in higher cell densities in cultures of these two strictly density-inhibited cell lines. In addition, when we transformed NIH3T3 cells by cultivation in fetal bovine serum, cells over-expressing human PiT1 formed more colonies in soft agar than control cells.

**Conclusions**

We conclude that not only is a certain level of PiT1 necessary for normal cell division as suggested by previously published studies, rather the cellular PiT1 level is involved in regulating cell proliferation and cell density and an increased PiT1 expression can indeed make NIH3T3 cells more sensitive to transformation. We have thus provided the first evidence for that expression of the type III P_i _transporter, PiT1, above the endogenous level can drive cell proliferation and overrule cell density constraints, and the results bridge previous observations showing that a certain PiT1 level is important for regulating normal embryonic growth/development and for tumorigenicity of HeLa cells.

## Background

The mammalian type III sodium-dependent inorganic phosphate (NaP_i_) symporters, PiT1 (SLC20A1; formerly GLVR1) and PiT2 (SLC20A2; formerly GLVR2 and Ram1) [1-6] belong to the P_i _transport (PiT) family (SLC20; TC #2.A.20 [7]), which members are present in all kingdoms of life [8]. The mammalian PiT proteins were originally identified as receptors for gammaretroviruses [2,3,5]. Thus, e.g., human PiT1 (hPiT1) is receptor for gibbon ape leukemia virus (GALV) [3] and feline leukemia virus subgroup B (FeLV-B) [9] and human PiT2 (hPiT2) and murine PiT2 (mPiT2) are receptors for amphotropic murine leukemia virus (A-MLV) [1,5]. There are, however, differences between the receptor functions of PiT proteins from different species, e.g., unlike hPiT1, murine PiT1 (mPiT1) does not support infection by GALV and FeLV-B [3,10-14].

Analysis of the P_i _transport functions of the two human PiT paralogs shows comparable abilities to transport P_i _[15]. Moreover, both paralogs are ubiquitously expressed in mammalian cells and have been suggested to be housekeeping P_i _transporters supplying cells with their general P_i _needs [1,16-18] and thus to have overlapping functions in cellular P_i _import. This notion is supported by results obtained by Beck and co-workers on an allelic series of mutant mice in which PiT1 was expressed from 0 to 100% [19]. In agreement with results from Festing and co-workers, they found that PiT1 knock-out mice did not survive past E12.5 [19,20]. Beck and co-workers, however, also found that E11.5 embryos showing PiT1 mRNA levels below 15% of that observed in wildtype embryos, had up-regulated PiT2 expression. And the authors could indeed not observe differences in the P_i _uptake abilities of mouse embryonic fibroblasts (MEFs) derived from wildtype and PiT1-knock-out embryos [19]. These results suggest that PiT2 might be compensating for the loss of PiT1 as a supplier, at least in part, of P_i _to the embryonic cells up to E11.5 to E12.5. However, despite these suggested overlapping functions of PiT1 and PiT2 in cellular P_i _import, Beck and co-workers also showed that PiT1 possesses a cellular function, which cannot be replaced by PiT2. Thus compared to wildtype embryos, at E10.5 PiT1-knock-out embryos started to show retarded growth and when investigated at E11.5 and/or E12.5, the PiT1 knock-out embryos also showed reduced proliferation of liver cells [19]. Moreover, although MEF cells derived from PiT1-knock-out embryos and from embryos showing PiT1 mRNA levels at 50% of wildtype levels had unimpaired P_i _uptake abilities, they did show severely impaired proliferation [19]. These observations suggest a function of PiT1 in proliferation, which is not related to cellular P_i _uptake. In line with this, the same group has also shown that knock-down of the expression of PiT1 in the two transformed human cell lines, HeLa and HepG2, severely impairs their proliferation [21]. In addition, HeLa cells with knocked down PiT1 expression showed severely reduced tumor growth after injection into nude mice compared to control HeLa cells [21]. The impaired proliferation of HeLa cells could not be rescued by over-expression of PiT2 although the latter restored cellular P_i _uptake to nearly wildtype levels. However, expression of a PiT1 mutant unable to transport P_i _did reestablish the proliferative potential of the PiT1-knock-down cells [21]. Thus, the results from embryos and MEF and HeLa cells suggest that the role of PiT1 in cell proliferation is not related to PiT1 P_i _uptake. Interestingly, studying MEF cells with either knocked out PiT1 expression or PiT1 mRNA levels at 50% of wildtype levels, Beck and co-workers found that the proliferative potentials of these cells correlated with the levels of PiT1 expression [19]. Thus, the PiT1-knock-out MEFs proliferated very slowly (2.1-fold decrease in doubling time compared to wildtype), while the proliferative potential of the MEFs with a 50% reduction in PiT1 mRNA levels was between those of the knock-out and wildtype MEFs [19]. The observations that lowering of the cellular PiT1 level led to reduced proliferation of different cell types, and of murine as well as human cells, [19,21] suggest that mPiT1 and hPiT1 have the same function in cell proliferation and that a certain level of PiT1 in general is necessary for normal cell proliferation. In addition, these observations also open for the possibility that the PiT1 level *per se* might be involved in controlling cell proliferation as hypothesized by Beck and co-workers [21]. In agreement with this hypothesis, upon establishing and cultivating murine MC3T3-E1 cells over-expressing hPiT1, we noticed that the hPiT1 expressing cells grew to higher densities than control cells albeit that the MC3T3-E1 cell line exhibits strictly density-inhibited proliferation (unpubl. observation).

We have here investigated the role of PiT1 in governing proliferation and cell density of two strictly density-inhibited cell lines, the murine MC3T3-E1 [22] and NIH3T3 [23] cells. In order to investigate whether an increased PiT1 level could influence the proliferation of these cells, we exploited, as elaborated above, that previous results suggest that mPiT1 and hPiT1 have the same function in cell proliferation and used MC3T3-E1 and NIH3T3 cells stably expressing hPiT1 for our experiments. This approach also allowed for verification of the presence of functional transgenic hPiT1 protein at the cell surface by exploiting the differences in retroviral receptor functions of mPiT1 and hPiT1. Furthermore, it allowed us to discriminate between exogenously and endogenously expressed hPiT1 and mPiT1 mRNAs, respectively, and thus to follow the mRNA levels of the endogenous mPiT1 in murine cells stably expressing hPiT1. Using a combination of PiT1 knock-down and hPiT1 over-expression studies, we found that the level of PiT1 in cells exhibiting density-inhibited growth, can determine their proliferative potential and the density to which these cells can grow. Specifically for both cell lines, over-expression of hPiT1 led to increased proliferation and cell density showing that a PiT1 level above the endogenous level can drive cell proliferation and to some degree overrule the cell density constraints of these strictly density-inhibited cell lines. Moreover, upon investigating their ability to form colonies in soft agar, we also found that over-expression of hPiT1 made NIH3T3 cells more sensitive to transformation with fetal bovine serum (FBS). We, furthermore, found that the endogenous PiT1 expression is regulated in a manner which indeed is in agreement with a role of PiT1 in controlling cell proliferation in density-inhibited cells.

## Results

### Knock-down of PiT1 impairs overall proliferation and cell density in cultures of MC3T3-E1 cells

**Figure 1:**
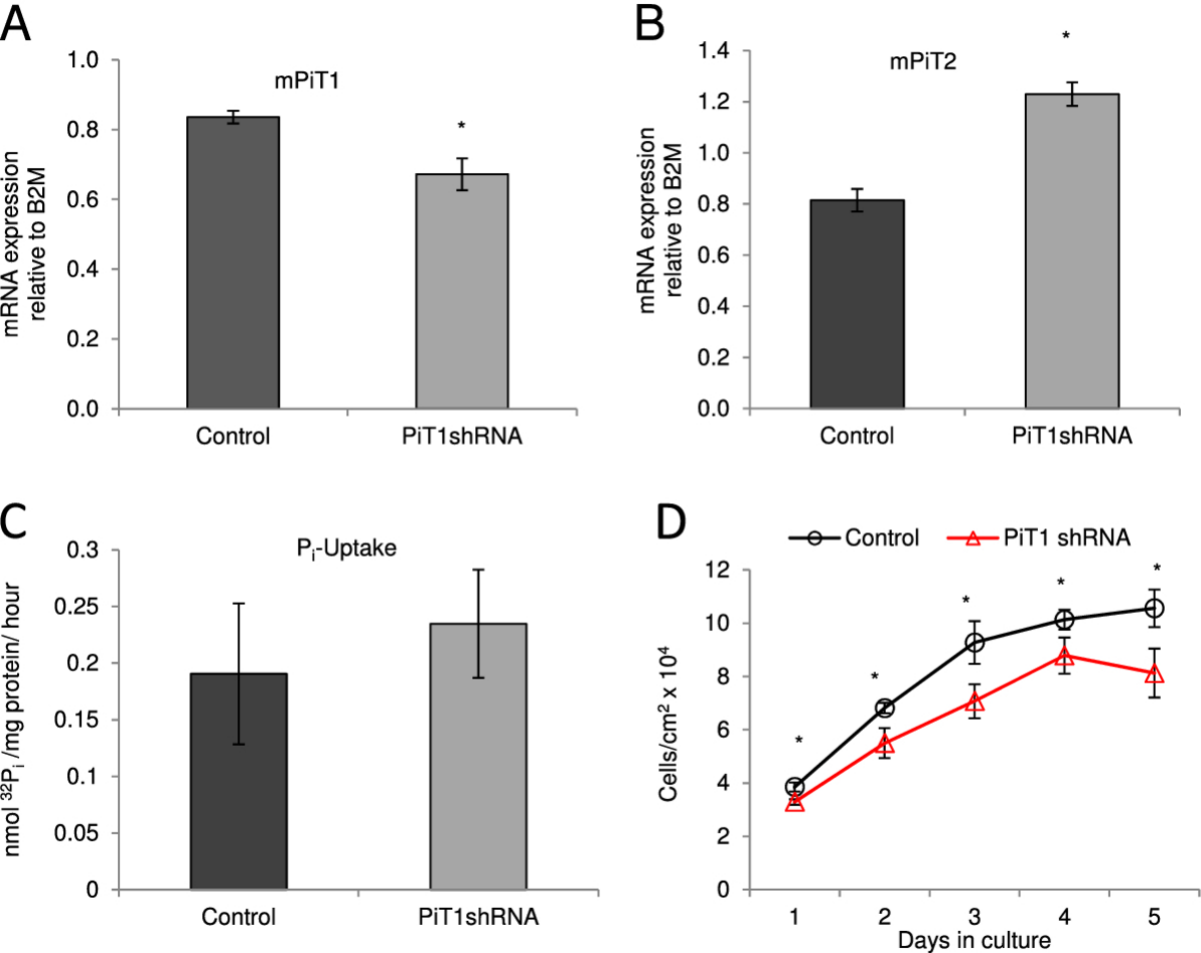
**Knock-down of PiT1 in MC3T3-E1 cells decreases overall proliferation and cell density**. A and B) qRT-PCR analyses. MC3T3-E1-PiT1shRNA (PiT1shRNA) and MC3T3-E1-LMP (Control) cells were seeded at 20,000 cells/cm^2 ^and after 2 days in culture, the mPiT1 (A) and mPiT2 (B) mRNA levels were analyzed by qRT-PCR. Each column represents cell lysates from three wells and triplicate qRT-PCR analyses of each cell lysate. The mPiT1 and mPiT2 mRNA levels are standardized to B2M mRNA levels. Data are means ± standard deviation (SD).*indicates statistically significantly different from control cells, p < 0.05. C) P_i_-transport assay. MC3T3-E1-PiT1shRNA (PiT1shRNA) and MC3T3-E1-LMP (Control) cells were seeded at 50,000 cells/cm^2 ^in 4-well plates. Two days after, ^32^P_i _import in P_i_-free medium (with 5 μM ^32^P_i _only) was analyzed over 5 minutes. The results are shown as mean ^32^P_i _import per mg protein per hour of 4 wells ± SD. D) Cell counts. MC3T3-E1-PiT1shRNA (PiT1shRNA) and MC3T3-E1- LMP (Control) cells were seeded at 20,000 cells/cm^2 ^in 4-well plates and counted at the indicated days. The results are shown as mean cell number per cm^2 ^of 4 wells ± SD.* indicates statistically significantly different from control cells at the same day, p < 0.05.

The pre-osteoblastic cell line, MC3T3-E1, was established following a 3T3 cultivation scheme [22] and maintains strictly density-inhibited proliferation in our laboratory when grown under conditions not inducing differentiation (unpubl. observation). We investigated how knock-down of the endogenous PiT1 (mPiT1) level affected proliferation of this strictly density-inhibited cell line. MC3T3-E1 cells with stable knock-down of PiT1 were made by transduction with a retroviral vector encoding a miR-based shRNA against mPiT1, and the cells are referred to as MC3T3-E1-PiT1shRNA. Compared to control cells transduced with the empty transfer vector (MC3T3-E1-LMP), MC3T3-E1-PiT1shRNA cells showed about 20% knock-down of the mPiT1 mRNA level (Figure 1A), and about 50% upregulation of the mPiT2 mRNA level (Figure 1B). The PiT1 knock-down did not influence the ability of the cells to import P_i _(Figure 1C). When MC3T3-E1-PiT1shRNA and MC3T3-E1-LMP cells were seeded at 20,000 cells/cm^2 ^and counted each day over 5 days, we observed that MC3T3-E1-PiT1shRNA cultures in general showed decreased proliferation and did not grow as dense as control (MC3T3-E1-LMP) cultures (Figure 1D). Moreover, in agreement with the importance of a certain PiT1 level for cell proliferation, we experienced difficulties in maintaining larger PiT1 knock-down levels in the stably transduced cultures (unpubl. observations). Thus a certain level of PiT1 was found to be important for proliferation of MC3T3-E1 cells as has previously been shown for MEFs and two human cancer cell lines [19,21]. Moreover, as observed for MEFs with down-regulated PiT1 expression [19], knock-down of PiT1 expression in MC3T3-E1 cells led to upregulation of the PiT2 expression, but no change in P_i _uptake.

### The endogenous PiT1 mRNA level is decreased in dense MC3T3-E1 cell cultures

**Figure 2:**
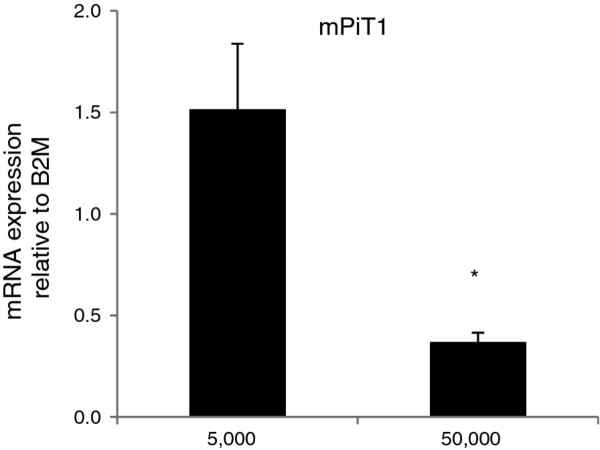
**Expression of endogenous PiT1 in MC3T3-E1 cells**. MC3T3-E1 cells were seeded at 5,000 and 50,000 cells/cm^2 ^in 4-well plates and 2 days after seeding, the mRNA levels of mPiT1 were analyzed by qRT-PCR. Each column represents cell lysates from three wells and triplicate qRT-PCR analyses of each cell lysate. The mPiT1 mRNA levels are standardized to B2M mRNA levels. Data are means ± SD.* indicates statistically significantly different from MC3T3-E1 cells seeded at 5,000 cells/cm^2^, p < 0.05.

The proliferation of the MC3T3-E1-PiT1shRNA cells with knocked down PiT1 expression seemed to decline from day 4 in culture while the control cultures had not entirely stopped proliferating at days 4 and 5 (Figure 1D). Interestingly, similar differences in the proliferative patterns were observed between MEF cells with decreased PiT1 expression and control cells [19]. This is intriguing since in either experiment had the cells with lowered PiT1 expressions, at the time they stopped proliferating, not reached the density of the control cultures (Figure 1D and ref. [19]). We have previously observed that the endogenous PiT1 mRNA level decreases in MC3T3-E1 cells over time in culture, i.e., as the cultures grow denser (unpubl. observations). To investigate whether the PiT1 expression was down-regulated as a consequence of density or time in culture, MC3T3-E1 cells were seeded at 5,000 cells/cm^2 ^(sparsely) or 50,000 cells/cm^2 ^(densely) and two days later, the PiT1 mRNA levels were analyzed (Figure 2). The cells seeded sparsely had more than 4 times higher PiT1 mRNA levels compared to densely seeded cells; thus the mPiT1 mRNA levels in the MC3T3-E1 cell line correlate with cell density rather than time in culture, with less dense cultures showing higher mPiT1 mRNA levels than denser cultures. Together with the observed ceased proliferation of the MC3T3-E1 (Figure 1D) and MEF [19] cells with lowered PiT1 levels, despite the capacity of the control cultures to grow denser, these results may suggest that the PiT1 level also influences how dense the cells can grow.

### Characterization of MC3T3-E1 cells over-expressing hPiT1 or hPiT2

The observation that knock-down of the PiT1 mRNA level in human [21] and murine (Figure 1D and ref. [19]) cells led to impaired proliferation and cell density strongly suggest that PiT1 has the same function in cell proliferation in mouse and man. In order to further address the role of the PiT1 level in cell proliferation and cell density, we therefore employed MC3T3-E1 cells stably expressing hPiT1. The use of hPiT1 allowed us both to investigate whether the transgenic hPiT1 protein was present in the cell membrane and to discriminate between endogenous and exogenous PiT1 mRNA levels in the cells.

**Figure 3:**
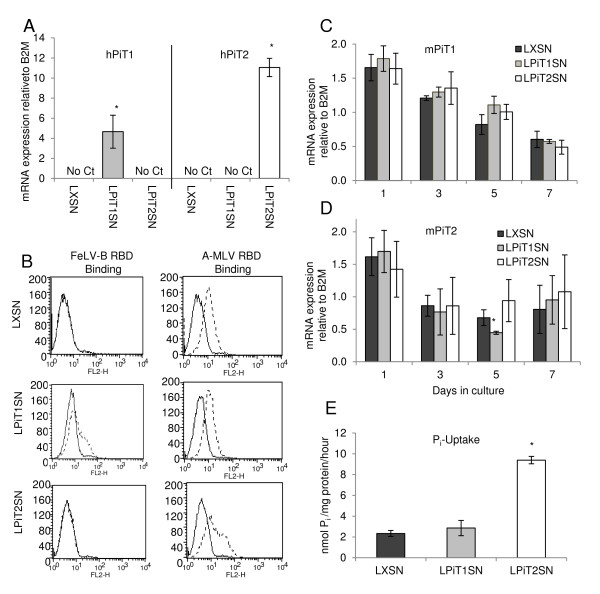
**Characterization of MC3T3-E1 cells over-expressing hPiT1 or hPiT2**. A) qRT-PCR analysis of exogenously expressed hPiT1 and hPiT2 mRNA levels in MC3T3-E1-LXSN (LXSN), MC3T3-E1-LPiT1SN (LPiT1SN), and MC3T3-E1-LPiT2SN (LPiT2SN) cells, respectively. Each column represents cell lysates from three wells and triplicate qRT-PCR analyses of each cell lysate. The hPiT1 and hPiT2 mRNA levels are standardized to the endogenous B2M mRNA levels. Data are means ± SD. "No CT" indicates that no signal was obtained for the transgene examined. * indicates statistically significantly different from MC3T3-E1-LXSN (control) cells, p <0.05. B) RBD-binding assay. MC3T3-E1-LXSN (LXSN), MC3T3-E1-LPiT1SN (LPiT1SN), and MC3T3-E1-LPiT2SN (LPiT2SN) cells were incubated with His-tagged FeLV-B RBD or His-tagged A-MLV RBD containing supernatant (dashed line) or in standard growth medium containing no RBD (solid line) followed by a mouse-anti-His antibody and finally a PE-conjugated goat anti-mouse Ig antibody. The cells were analyzed by flow cytometry. The x-axis shows PE intensity measured in the FL2-H channel. C and D) Expression of endogenous mPiT1 and mPiT2. MC3T3-E1-LXSN (LXSN), MC3T3-E1-LPiT1SN (LPiT1SN), and MC3T3-E1-LPiT2SN (LPiT2SN) cells were seeded at 500 cells/cm^2 ^in 6-well plates and the endogenous mPiT1 mRNA levels were analyzed by qRT-PCR at the indicated days in culture. Each column represents six wells, but since the wells were harvested two and two in the same cell lysis buffers, the columns represent three lysates, and triplicate qRT-PCR analyses of each cell lysate. The mPiT1 and mPiT2 mRNA levels are standardized to B2M mRNA levels. Data are means ± SD. E) P_i_-transport assay. MC3T3-E1-LXSN (LXSN), MC3T3-E1-LPiT1SN (LPiT1SN), and MC3T3-E1-LPiT2SN (LPiT2SN) cells were seeded at 20,000 cells/cm^2 ^in 4-well plates. The next day, ^32^P_i _import in medium containing a total [P_i_] of 100 μM was analyzed over 5 minutes. The results are shown as mean ^32^P_i _import per mg protein per hour of 4 wells ± SD. * indicates statistically significantly different from MC3T3-E1-LXSN (control) cells, p < 0.05.

We established populations of MC3T3-E1 cells stably expressing hPiT1 (MC3T3-E1-LPiT1SN), hPiT2 (MC3T3-E1-LPiT2SN), or empty transfer vector (MC3T3-E1-LXSN) by retroviral transduction. The transgenic hPiT1 and hPiT2 were found to be expressed in MC3T3-E1-LPiT1SN and -LPiT2SN cells, respectively, at the mRNA level by quantitative RT-PCR (qRT-PCR) analysis (Figure 3A). We exploited the retroviral receptor functions of hPiT1 and hPiT2 to analyze for their presence on the cell surface using binding assays employing the receptor binding domains (RBDs) of the two viruses FeLV-B and A-MLV, respectively. FeLV-B can use hPiT1 but not mPiT1 as receptor and, in agreement with this, MC3T3-E1-LXSN cells did not bind FeLV-B RBD, while MC3T3-E1-LPiT1SN cells bound FeLV-B RBD showing that hPiT1 was present at the surface of these cells (Figure 3B). MC3T3-E1-LPiT2SN cells did not show increased FeLV-B binding compared to MC3T3-E1-LXSN cells, in agreement with that hPiT2 does not bind FeLV-B (Figure 3B). As well mPiT2 as hPiT2 are receptors for A-MLV, accordingly, MC3T3-E1-LXSN cells showed A-MLV RBD binding, however, MC3T3-E1-LPiT2SN cells exhibited increased A-MLV RBD binding showing that hPiT2 was present at the surface of these cells (Figure 3B). MC3T3-E1-LPiT1SN cells did not show increased A-MLV binding compared to MC3T3-E1-LXSN cells, showing that expression of hPiT1 did not lead to increased expression of endogenous mPiT2 (Figure 3B). The surface expression of functional hPiT1 and hPiT2 can be analyzed by investigating the transduction of the cells by retroviral vectors carrying surface proteins of viruses, which can use the proteins as receptors. We here used GALV and A-MLV pseudotyped GBN transfer vectors, where GBN encodes β-galactosidase. GALV can, as FeLV-B, use hPiT1 but not mPiT1 as a receptor (Table 1). The exogenously expressed hPiT1 and hPiT2 proteins were functionally expressed at the cell membrane of MC3T3-E1-LPiT1SN and MC3T3-E1-LPiT2SN, respectively, in that MC3T3-E1-LPiT1SN cells were susceptible to transduction with GALV pseudotyped GBN vectors and MC3T3-E1-LPiT2SN cells showed increased transduction with A-MLV pseudotyped GBN vectors compared to MC3T3-E1-LPiT1SN and control (MC3T3-E1-LXSN) cells (Table 1).

**Table 1:** Transduction study analyzing the presence of functional hPiT1, hPiT2, and mPiT2 on the cell surface of MC3T3-E1-LXSN, -LPiT1SN, -LPiT2SN, NIH3T3-LXSN, and - LPiT1SN cells

Cells ^a)^	Pseudotype of vectors encoding LacZ	
	
	A-MLV ^b)^	GALV
MC3T3-E1-LXSN	4.7 × 10^4 c)^	0 ^d)^

MC3T3-E1-LPiT1SN	4.5 × 10^4^	4.3 × 10^3^

MC3T3-E1-LPiT2SN	6.5 × 10^4^	0

NIH3T3-LXSN	6.4 × 10^5^	0

NIH3T3-LPiT1SN	5.4 × 10^5^	3.6 × 10^4^

a) MC3T3-E1 cells transduced with empty vector (LXSN), LPiT1SN, or LPiT2SN retroviral vectors were challenged with retroviral vectors pseudotyped with A-MLV or GALV surface proteins and carrying a β-galactosidase encoding transfer vector.

b) The titers on D17 cells of the vector stocks used were 1.7 × 10^5 ^CFU/mL (A-MLV pseudotype) and 1.9 × 10^5 ^CFU/mL (GALV pseudotype).

c) Numbers indicate blue CFU per mL vector containing supernatant.

d) No blue cells were detected in the wells.

The endogenous mPiT1 and mPiT2 mRNA levels were also followed over time in culture in MC3T3-E1-LXSN, -LPiT1SN, and -LPiT2SN cells. In the experiment shown in Figures 3C and 3D, the cells were seeded at 500 cells/cm^2^, thus the cells grew in colonies. In agreement with the observed regulation of endogenous mPiT1 in wildtype MC3T3-E1 cells, the mPiT1 mRNA levels decreased when the cells started to touch each others within the colonies, i.e., approximately around 3-5 days in culture in the experiment shown (Figure 3C). No significant difference in mPiT1 mRNA levels was observed between MC3T3-E1-LXSN, -LPiT1SN or -LPiT2SN cells (Figure 3C) indicating that the expression of the endogenous mPiT1 was not *per se* influenced by the exogenously expressed hPiT1 or hPiT2. The endogenous mPiT2 mRNA levels decreased from day 1 to 3 in culture and remained thereafter at the same level (Figure 3D). At day 5 in culture, MC3T3-E1-LPiT1SN cells showed lower mPiT2 mRNA levels than the other cultures, but otherwise, no significant difference in mPiT2 mRNA levels was observed between MC3T3-E1-LXSN, -LPiT1SN or -LPiT2SN cells (Figure 3D) indicating that the expression of the endogenous mPiT2 was not *per se* influenced by the exogenously expressed hPiT1 or hPiT2.

### Over-expression of hPiT1 in MC3T3-E1 cells increases cell proliferation and cell density

**Figure 4:**
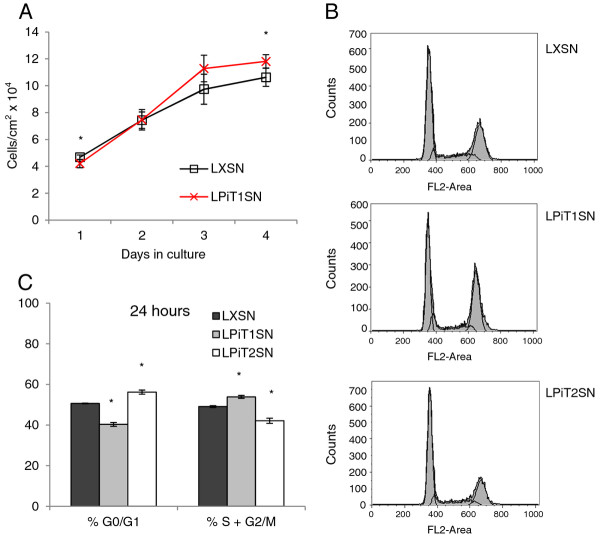
**Over-expression of hPiT1 increases proliferation and cell density of MC3T3-E1 cultures**. A) Cell counts. MC3T3-E1-LPiT1SN (LPiT1SN) (crosses) and control cells (MC3T3-E1-LXSN (LXSN)) (squares) cells were seeded at 20,000 cells/cm^2 ^in 4-well plates and counted at the indicated days. The results are shown as mean cell number per cm^2 ^of 4 wells ± SD. *indicates statistically significantly different from MC3T3-E1-LXSN cells at the same day, p <0.05. B and C) Cell cycle analysis. MC3T3-E1-LPiT1SN (LPiT1SN), MC3T3-E1-LXSN (LXSN), and MC3T3-E1-LPiT2SN (LPiT2SN) were seeded at 20,000 cells/cm^2 ^in T25-flasks, stained with propidium iodide 24 hours later, and analyzed by flow cytometry. B) Representative flow cytometry histograms of MC3T3-E1-LXSN (LXSN) (top), MC3T3-E1-LPiT1SN (LPiT1SN) (middle), and MC3T3-E1-LPiT2SN (LPiT2SN) (bottom) cells; the thick black lines represent the Watson-Pragmatic model. C) The percentages of cells in the respective phases of the cell cycle obtained using the Watson Pragmatic model. Each column represents the mean of triplicate set ups ± SD. *indicates statistically significantly different from MC3T3-E1-LXSN (control) cells in the same phases of the cell cycle, p < 0.05.

To further address the importance of the PiT1 level in cell proliferation and cell density, we employed the hPiT1 expressing MC3T3-E1-LPiT1SN cells to investigate whether increased PiT1 expression levels could influence the proliferation of the MC3T3-E1 cell line. In order to be able to follow the possible effects of hPiT1 over-expression on cell proliferation and cell density, and at the same time allowing a uniform cell density in the cultures, we seeded MC3T3-E1-LPiT1SN cells and, as control, MC3T3-E1-LXSN cells at densities of 20,000 cells/cm^2 ^(Figure 4A). We found that cultures of the hPiT1 expressing MC3T3-E1-LPiT1SN cells showed increased proliferation until day 3 in culture and, in agreement with our previous observations, achieved a higher cell density after 4 days in culture than cultures of control MC3T3-E1-LXSN cells (11.8 × 10^4 ^cells/cm^2 ^compared to 10.6 x 10^4 ^cells/cm^2^, respectively, in the experiment shown in Figure 4A). This observation is not trivial since MC3T3-E1 cells show strictly density-inhibited proliferation. We also analyzed the influence of over-expression of hPiT1 on the distribution of cells in the G0/G1, S, and G2/M phases of the cell cycle at day 1 in culture in independent set-ups (Figures 4B and 4C). In agreement with the increased proliferative potential of the hPiT1 expressing MC3T3-E1-LPiT1SN cells, cultures of these had a statistically significantly higher number of cells in S + G2/M compared to control cultures (53.9 ± 0.7 compared to 49.1 ± 0.5, respectively). Thus, if the PiT1 level is kept at a higher level than in control cells by exogenous expression of hPiT1 in the strictly density-inhibited MC3T3-E1 cells, the cells show initially higher proliferation and can grow to a higher density than control cells. Unlike MC3T3-E1-LPiT1SN cells, cultures of MC3T3-E1 cells expressing hPiT2, MC3T3-E1-LPiT2SN, did not show increased number of cells in S + G2/M phases compared to control cultures (Figures 4B and 4C). To summarize, a certain PiT1 level is not only important for proliferation of MC3T3-E1 cells, an increase in the PiT1 level can impel proliferation and to some degree overrule the constraints on cell density.

### Effect of regulation of PiT1 and PiT2 expressions on the ability of MC3T3-E1 cells to import P_i_

The impaired proliferation of MC3T3-E1 (Figure 1D) and the previously reported impaired proliferation of MEF cells [19], as consequences of knock-down of PiT1 expression, were both associated with upregulated PiT2 expression and neither could be related to decreased cellular P_i _import. Moreover, the previously reported down-regulatory effect of knock-down of PiT1 on proliferation of HeLa cells was shown to be independent on the ability of PiT1 to transport P_i _[21]. We, moreover, find that when seeded at a density of 50,000 cells/cm^2^, or 20,000 cells/cm^2 ^as in the experiment shown in Figure 4, analyses of the P_i _uptake as in the experiment shown in Figure 1C show that the ability of MC3T3-E1-LPiT1SN cells to import P_i _is indistinguishable from that of MC3T3-E1-LXSN cells, while that of MC3T3-E1-LPiT2SN cells is several fold higher (unpubl. results). The Km's of hPiT1 and hPiT2 are in the range of 100 to 300 μM P_i _[15], and the P_i _uptake was also analyzed in the presence of 100 μM and 300 μM P_i_. However, no increase in P_i _uptake by the MC3T3-E1-LPiT1SN cells compared to control MC3T3-E1-LXSN cells was observed, neither at 100 μM P_i _(Figure 3E) nor at 300 μM P_i _(not shown); MC3T3-E1-LPiT2SN cells showed 4-fold (100 μM P_i_) (Figure 3E) and 3.3-fold (300 μM P_i_) (not shown) increased P_i _uptake compared to control MC3T3-E1-LXSN cells. The lack of increased P_i _import in the PiT1 over-expressing MC3T3-E1 cells are in support of that the effects of the PiT1 level on the proliferation and density of MC3T3-E1 cells could be independent of its P_i_-transport function. Further supporting that the cellular P_i _import *per se* is not the cause of the effect of the increased PiT1 level on the proliferative potential of MC3T3-E1 cells is that MC3T3-E1-LPiT2SN cells, which exhibited increased P_i _uptake ability, showed the opposite pattern of distribution of cells in G0/G1 and S + G2/M than MC3T3-E1-LPiT1SN cells (Figure 4C).

### Characterization of NIH3T3 cells over-expressing hPiT1

**Figure 5:**
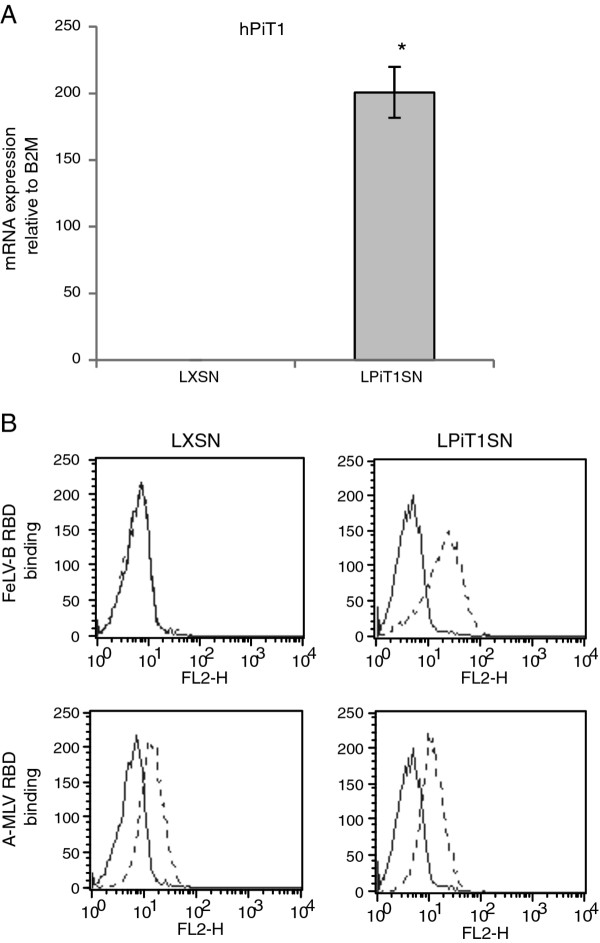
**Characterization of NIH3T3 cells over-expressing hPiT1**. A) qRT-PCR analysis of exogenously expressed hPiT1 in NIH3T3-LPiT1SN (LPiT1SN) and NIH3T3-LXSN (LXSN) cells. Each column represents cell lysates from three wells and triplicate qRT-PCR analyses of each cell lysate. The hPiT1 mRNA levels are standardized to the endogenous B2M mRNA levels. Data are means ± SD.* indicates statistically significantly different from NIH3T3-LXSN (control) cells. B) RBD-binding assay. Analyses of binding of His-tagged FeLV-B RBD and A-MLV RBD to NIH3T3-LXSN (LXSN) and NIH3T3-LPiT1SN (LPiT1SN) cells were performed as described in the legend to Figure 3. Incubations with His-tagged FeLV-B RBD containing supernatant (dashed line) or standard growth medium (solid line) are shown. The x-axis shows PE intensity measured in the FL2-H channel.

To address whether the effects of hPiT1 over-expression on regulation of cell proliferation and density of MC3T3-E1 cells are cell-type specific and/or are a result of MC3T3-E1 being a pre-osteoblastic cell line, for which PiT1 is important in differentiation/mineralization (unpubl. results) [24,25], we wished to investigate the effects of hPiT1 over-expression on the murine fibroblastic cell line, NIH3T3. The NIH3T3 cell line, like the MC3T3-E1 cell line, was established following a 3T3 cultivation scheme [23]; it maintains strictly density-inhibited proliferation in our laboratory when cultivated in newborn calf serum (unpubl. observation). Populations of NIH3T3 cells that stably express hPiT1 (NIH3T3-LPiT1SN) or empty vector (NIH3T3-LXSN) were established by retroviral transduction. NIH3T3-LPiT1SN cells were shown to express hPiT1 at the mRNA level by qRT-PCR analysis (Figure 5A). The presence of the hPiT1 proteins on the cell surface was analyzed using the binding assay employing FeLV-B RBD, which as mentioned above, can bind hPiT1 but not mPiT1. As expected, NIH3T3-LXSN cells did not show binding of FeLV-B RBD, however, NIH3T3-LPiT1SN cells bound FeLV-B RBD showing that the hPiT1 protein was present at the surface of these cells (Figure 5B). Furthermore, we found that NIH3T3-LPiT1SN cells were susceptible to transduction with GALV pseudotyped GBN vector, and since GALV can only use hPiT1 and not mPiT1 as a receptor, this shows that the exogenously expressed hPiT1 protein was functionally expressed at the cell surface of the NIH3T3-LPiT1SN cells (Table 1). The endogenous mPiT2 is as mentioned above a receptor for A-MLV and in agreement with this, NIH3T3-LXSN cells showed A-MLV RBD binding (Figure 5B) and were transduced with A-MLV pseudotyped vectors (Table 1). NIH3T3-LPiT1SN cells did, however, not show increased A-MLV-specific binding (Figure 5B) or transduction (Table 1) compared to NIH3T3-LXSN cells, showing that expression of hPiT1 did not lead to increased expression of endogenous mPiT2 at the cell surface.

### Over-expression of hPiT1 in NIH3T3 cells increases cell proliferation and cell density

**Figure 6:**
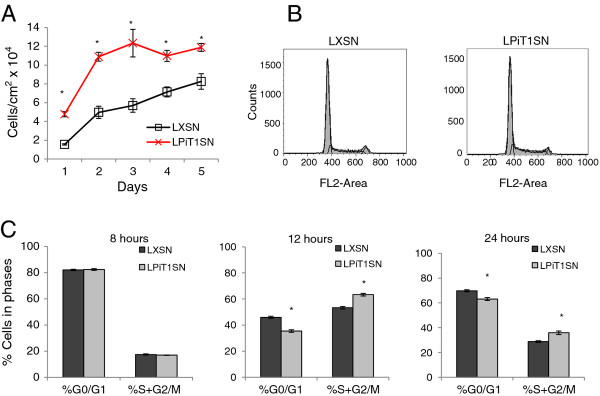
**Over-expression of hPiT1 increases proliferation and cell density of NIH3T3 cultures**. A) Cell counts. Control (NIH3T3-LXSN (LXSN)) (squares) and NIH3T3-LPiT1SN (LPiT1SN) (crosses) cells were seeded at 20,000 cells/cm^2 ^in 4-well plates and counted at the indicated days. The results are shown as mean cell number per cm^2 ^of 4 wells ± SD. *indicates statistically significantly different from NIH3T3-LXSN cells at the same day, p < 0.05. B and C) Cell cycle analysis. NIH3T3-LPiT1SN (LPiT1SN) and NIH3T3-LXSN (LXSN) were seeded at 20,000 cells/cm^2 ^in T25-flasks, and cultures were stained with propidium iodide 8, 12, and 24 hours later, and analyzed by flow cytometry. B) Representative flow cytometry histograms of NIH3T3-LXSN (LXSN) (left) and NIH3T3-LPiT1SN (LPiT1SN) (right) cells at 24 hours after seeding; the thick black lines represent the Watson-Pragmatic model. C) The percentages of cells in the respective phases of the cell cycle 8, 12, and 24 hours after seeding as indicated obtained using the Watson Pragmatic model. Each column represents the mean of triplicate set ups ± SD. *indicates statistically significantly different from NIH3T3-LXSN (control) cells in the same phases of the cell cycle, p < 0.05.

The effects of expressing hPiT1 on proliferation and density of NIH3T3 cells were investigated by seeding NIH3T3-LPiT1SN and, as control, NIH3T3-LXSN cells at densities of 20,000 cells/cm^2 ^and following the cell numbers each day over 5 days in culture (Figure 6A). Cultures of the hPiT1 expressing NIH3T3-LPiT1SN cells showed increased proliferation the first two days in culture and were able to grow denser compared to cultures of control NIH3T3-LXSN cells (e.g., 11.9 × 10^4 ^cells/cm^2 ^compared to 8.26 × 10^4 ^cells/cm^2^, respectively, after 5 days in culture in the experiment shown in Figure 6A). We also analyzed the influence of over-expression of hPiT1 on the distribution of cells in the G0/G1, S, and G2/M phases of the cell cycle 8, 12, and 24 hours after seeding in an independent set-up (Figures 6B and 6C). At 8 hours after seeding, there was no difference in the distribution of cells in the G0/G1 and S + G2/M phases of the cell cycle between hPiT1 over-expressing cells and control cells. However, in agreement with their increased proliferative potential, NIH3T3-LPiT1SN cultures showed a statistically significantly higher number of cells in S + G2/M compared to control cultures at 12 (63.3 ± 0.8 compared to 53.3 ± 0.8, respectively) and 24 hours (35.9 ± 1.5 compared to 28.8 ± 0.8, respectively) after seeding. Thus in NIH3T3 cells, an increased PiT1 level can also drive proliferation and overrule the constraints on cell density.

**Figure 7:**
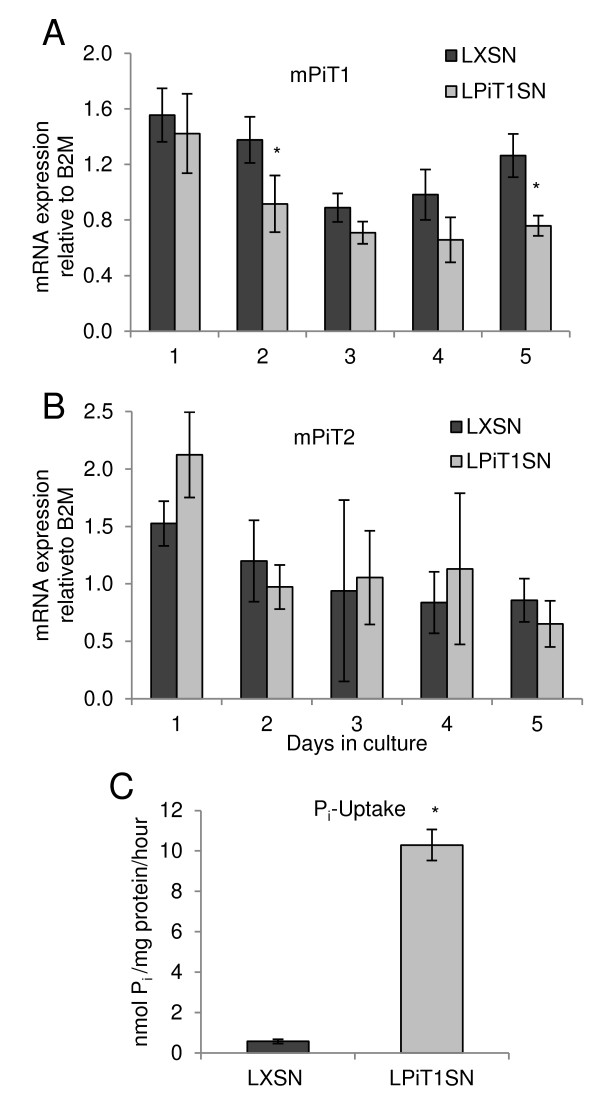
**Effect of over-expression of hPiT1 in NIH3T3 cells on the endogenous mPiT1 and mPiT2 mRNA levels and P_i _uptake**. A and B) qRT-PCR analyses of endogenously expressed mPiT1 and mPiT2. NIH3T3-LXSN (LXSN) and NIH3T3-LPiT1SN (LPiT1SN) cells were seeded at 20,000 cells/cm^2 ^in 4-well plates and the endogenous mPiT1 (A) and mPiT2 (B) mRNA levels were analyzed by qRT-PCR at indicated days in culture. Each column represents cell lysates from three wells and triplicate qRT-PCR analyses of each cell lysate. The mPiT1 and mPiT2 mRNA levels are standardized to B2M mRNA levels. Data are means ± SD. * indicates statistically significantly different from NIH3T3-LXSN (control) cells at the same day, p < 0.05. E) P_i_-transport assay. NIH3T3-LXSN (LXSN) and NIH3T3-LPiT1SN (LPiT1SN) cells were seeded at 20,000 cells/cm^2 ^in 4-well plates. The next day, ^32^P_i _import in medium containing a total [P_i_] of 100 μM was analyzed over 5 minutes. The results are shown as mean ^32^P_i _import per mg protein per hour of 4 wells ± SD. * indicates statistically significantly different from NIH3T3-LXSN (control) cells, p < 0.05.

In the experiment in Figure 6A, we also followed the endogenous mPiT1 and mPiT2 mRNA levels during the entire cultivation period (Figures 7A and 7B). Over-expression of hPiT1 led to a slight albeit not statistically significantly increased level of endogenous mPiT2 mRNA at day 1 in culture (compare NIH3T3-LXSN and NIH3T3-LPiT1SN at day 1 in Figure 7B), which however, in agreement with its non-significant size, was not reflected in the level of mPiT2 protein in the membrane (A-MLV binding assay in Figure 5B and A-MLV transduction assay in Table 1); no differences in the mRNA levels of endogenous mPiT2 were observed between NIH3T3-LXSN and NIH3T3-LPiT1SN cells at the remaining time points (Figure 7B). Over-expression of hPiT1 did not affect the endogenous mPiT1 mRNA levels, since NIH3T3-LPiT1SN and control NIH3T3-LXSN cells exhibited similar mPiT1 mRNA levels at day 1 in culture (Figure 7A). The proliferation of the control NIH3T3-LXSN cells varied over the entire cultivation period, but was lowest between days 2 and 3; interestingly, the endogenous mPiT1 level was lowest at day 3. The NIH3T3-LPiT1SN cells had stopped proliferating at day 2 in culture at a high cell density, and the mRNA level of endogenous mPiT1 was down-regulated at day 2 in these cells and stayed down to the end of the cultivation period (Figure 7A).

### Effect of over-expression of hPiT1 on the ability of NIH3T3 cells to import P_i_

The effect of over-expressing hPiT1 on the ability of NIH3T3 cells to import P_i _was also investigated. Unlike MC3T3-E1 cells, which did not support increased P_i _import when hPiT1 was over-expressed, the hPiT1 expressing NIH3T3-LPiT1SN cells were found to support increased (approximately 20-fold) P_i _import compared to control NIH3T3-LXSN cells (Figure 7C).

### Effect of over-expression of hPiT1 on the ability of NIH3T3 cells to form colonies in soft agar

Previous studies showed that down-regulation of the PiT1 level in HeLa cells impaired their ability to form tumors in nude mice [21]. In order to investigate whether it was possible to bridge these data with the here found role of increased PiT1 level on proliferation of density-inhibited cells, we exploited that part of the cells in density-inhibited NIH3T3 cultures can be transformed by cultivation in FBS. Thus, NIH3T3-LPiT1SN and control NIH3T3-LXSN cells were cultivated in FBS for 2 days and their abilities to form colonies in soft agar were investigated; as control, cultures cultivated in normal growth medium, i.e., containing NCS, were also included in the soft agar assay shown in Table 2. For both NIH3T3-LPiT1SN and control NIH3T3-LXSN, only the cultures cultivated in FBS for 2 days formed substantial numbers of colonies in soft agar, however, a statistically significant increase in the number of colonies was found in soft agar cultures of NIH3T3-LPiT1SN cells compared to NIH3T3-LXSN cells (97.5 ± 9.1 and 69.0 ± 8.3, respectively). Thus over-expression of hPiT1 made the NIH3T3 cells more sensitive to transformation with FBS.

**Table 2:** Colony formation by NIH3T3-LXSN and NIH3T3-LPiT1SN cells in soft agar

Cells	NCS ^a)^	FBS ^b)^
NIH3T3-LXSN	0.5 ± 0.5^c)^	69.0 ± 8.3

NIH3T3-LPiT1SN	2.5 ± 1.8	97.5 ± 9.8^d)^

a) The cells were cultured in normal growth medium prior to as well as during cultivation in soft agar (DMEM supplemented with 10% NCS and 1% PS).

b) The cells were cultured in transformation medium 2 days prior to as well as during cultivation in soft agar (DMEM supplemented with 10% FBS and 1% PS).

c) Numbers indicate mean numbers of colonies per well of 4 wells ± SD.

d) Statistically significantly different from colonies derived from NIH3T3-LXSN (control) cells grown in FBS, p < 0.05.

## Discussion

In the present work, we show that the type III NaP_i _transporter, PiT1, can regulate proliferation and cell density of two different non-transformed cell lines, MC3T3-E1 and NIH3T3, which both exhibit strictly density-inhibited cell proliferation, and that over-expression of PiT1 indeed made NIH3T3 cells more sensitive to transformation with FBS. Working with MC3T3-E1 cells, we found in agreement with the *in vitro* work of Beck and co-workers in MEF, HeLa, and HepG2 cells [19,21] and the reported effect of PiT1 knock-out on proliferation of liver cells in the developing embryo [19], that lowering of the PiT1 level led to impaired cell proliferation; interestingly, so far we have not identified a PiT1-knock-down level, which the NIH3T3 cells could survive (unpubl. observations). Thus, a certain PiT1 level is necessary for maintaining the wildtype proliferative potentials of a number of different cell lines/cell types. We, however, also found that over-expression of PiT1 increased the proliferation and culture densities of MC3T3-E1 and NIH3T3 cells and made NIH3T3 cells more sensitive to transformation with FBS. This is the first time that it is shown that an increase in the PiT1 expression above the endogenous levels of cells is sufficient to increase cell proliferation and that it indeed also can increase the density to which strictly density-inhibited cell lines can grow as well as increase anchorage independent growth/transformation. Thus, not only is a certain level of PiT1 necessary for normal cell proliferation (Figure 1 and refs. [19,21]), an increased PiT1 level above the endogenous level of the cell can impel proliferation and to some degree overrule the constraints on cell density. Hence, the results suggest that PiT1 is not only necessary for normal cell proliferation, but can regulate the proliferative potential of cells, cell density, and anchorage-independent growth.

Investigations of the endogenous PiT1 mRNA level in control MC3T3-E1 and NIH3T3 cells showed that it seemed to follow the proliferative potential of the cells, i.e., the lowest PiT1 mRNA levels were found in cultures showing a low degree of proliferation, in agreement with a role of the endogenous PiT1 level in proliferation. Our observations also suggest a role of the endogenous PiT1 level in regulating cell density. Thus we found that the endogenous PiT1 mRNA level was down-regulated in dense cultures. Moreover, while increasing the PiT1 levels in NIH3T3 and MC3T3-E1 cells by over-expressing hPiT1 did not lead to an obvious abrogation of the density-inhibited cell proliferation *per se*, the cultures did grow to higher cell densities than control cell cultures before they stopped proliferating. Furthermore, while the NIH3T3 cells over-expressing hPiT1 showed similar endogenous PiT1 mRNA levels as the control cells at day 1 in culture, at day 2 in culture, these cells had reached their maximal density and had down-regulated the endogenous PiT1 mRNA levels, which stayed down. It is possible that the down-regulation of endogenous PiT1 could have occurred at a lesser cell density than the maximal density of the hPiT1 over-expressing cultures, since over-expression of hPiT1 led to higher cell densities, than observed in control cultures, and we only had one analysis-point per day. Nevertheless, the observed down-regulation of the endogenous PiT1 expression in response to cell density is interesting in that the studies of MC3T3-E1 (Figure 1) and MEF [19] cells showed that down-regulation of their PiT1 mRNA levels was sufficient to down-regulate the proliferation of these cells and that they ceased to proliferate although they had not reached the density of the control cells. It is in this respect also interesting, that Beck and co-workers reported on a less dense packaging of cells in the liver of PiT1 knock-out embryos [19]. Together these data make us hypothesize that the cells themselves utilize their PiT1 levels to control their proliferation in response to cell density, i.e., when the cells attain a certain density, they down-regulate the endogenous PiT1 level, which again leads to down-regulation of the proliferation.

It is currently not known how the increased and decreased PiT1 levels lead to increased and decreased, respectively, cell proliferation. Beck and co-workers found that impairment of proliferation of HeLa cells by knock-down of endogenous PiT1 could be rescued by a PiT1 mutant (S128A mutation), which is unable to transport P_i_, but not by over-expression of PiT2 albeit the latter nearly restored the P_i _uptake ability of the cells to wildtype level [21]. Furthermore, Beck and co-workers also found that the impaired proliferation of MEF cells with down-regulated PiT1 expression was not due to impaired cellular P_i _uptake [19]. In the present work, did over-expression of hPiT1 not lead to increased P_i _uptake in MC3T3-E1 cells. However, over-expression of hPiT2 in these cells did lead to increased P_i _uptake ability, but not to an increased proportion of cells in the S + G2/M phases of the cell cycle as over-expression of hPiT1 did. Thus, we have no evidence supporting that the here shown function of PiT1 in cell proliferation in MC3T3-E1 cells is related to P_i _translocation by PiT1. However, since hPiT1 over-expression did increase the P_i_-uptake ability of NIH3T3 cells, we can presently not rule out that increased P_i _import play a role in NIH3T3 cells. Given the ubiquitous expression of PiT1 in mammalian cells [17], we do however hypothesize that the mechanism by which the PiT1 level exerts its function in the regulation of cell proliferation/cell density is the same in the different cell types.

In PiT1-deficient MEF cells, Beck and co-workers investigated the activation of S6K1, S6, Akt1, and 4EBP1, which are proteins involved in coupling signals from growth factors and nutrients to coordinate cell growth and cell division, but found no defects in their regulation [19]. It should, however, be stressed that numerous studies show that PiT1 is a genuine cellular P_i _transporter, i.e., that it is able to import P_i _into cells, hereunder studies in *Xenopus laevis *oocytes [1,4,15,18,26], as we also find when we over-express hPiT1 in NIH3T3 cells. Thus, although the role of PiT1 in regulating cell proliferation may not be directly linked to its P_i _transport function, we nevertheless hypothesize that the role of the PiT1 level in regulating cell proliferation, relates to PiT1's suggested role in cellular P_i _homeostasis albeit in a yet unknown manner. It is in this respect, however, interesting that the yeast P_i _transporter, Pho84, can function as a nutrient sensor, a transceptor [27]. Results obtained by Popova and co-workers suggest that binding of phosphate/phosphate-containing compounds to Pho84 can elicit a signal transduction. The Pho84-mediated signal transduction did require a specific conformational change as in a P_i_-transport-process, but not a complete transport cycle, thus P_i _translocation was not involved in the signal transduction [27]. Albeit, it is not known whether PiT1 has transceptor function, it is important to notice that if there is analogy to the transceptor function of Pho84, a PiT1 P_i _transport knock-out mutant, as, e.g., the PiT1 S128A mutation shown to be able to rescue the impairment of proliferation of HeLa cells caused by knock-down of endogenous PiT1 [21], could be captured in a constitutive signaling conformation.

Another question arises from the present and published results on the role of PiT1 as a regulator of cell proliferation/cell density, namely, whether the abilities of PiT1 to transport P_i _and function as a regulator of cell proliferation/cell density are functions that are performed by the same PiT1 molecule? It is in this respect interesting, that PiT1 has been published to be able to bind another ubiquitously expressed protein, the multimembrane-spanning protein, progressive ankylosis protein (ANK), which exports pyrophosphate out of the cell [28]. How this binding affects the function of PiT1, is, however, not yet clear.

## Conclusions

We conclude that not only is a certain level of PiT1 necessary for normal cell division as suggested by previous published results, rather the cellular PiT1 level is involved in regulating cell proliferation and cell density, and expression of PiT1 above the endogenous level can drive cell proliferation, overrule cell density constraints, and indeed make NIH3T3 cells more sensitive to transformation. We have thus provided the first evidence for a role of the type III P_i _transporter, PiT1, as a protein, which can increase normal cell proliferation and cell density.

## Methods

### Expression plasmids and transfer vectors

The expression plasmids pOJ74 and pOJ75 (Wyeth-Ayerst Research, Pearl River N.Y., USA) encoding hPiT2 and hPiT1, respectively, have been described [11]. The pLXSN [29] derivatives, pLPiT1SN and pLPiT2SN carrying the open reading frames from pOJ75 and pOJ74, respectively, were constructed as follows. HindIII-NotI fragments harboring the coding sequences of pOJ74 and pOJ75 were purified, blunt ended using Klenow Fragment, and inserted into the HpaI site of the pLXSN plasmid [29], encoding the retroviral transfer vector LXSN; the resulting plasmids, pLPiT1SN and pLPiT2SN, respectively, were verified by sequence analysis (Kjærgaard, M. M. and Pedersen, L., unpublished work). The LMP [30] (Open Biosystems, France) derived transfer vector, LMP-MmPiT1shRNAmiR, was made by insertion of a shRNA encoding sequence targeting murine PiT1: 5'TGCTGTTGAC AGTGAGCGAA **CCCATTGTAT TGTCGGTGC**A TAGTGAAGCC ACAGATGTAT **GCACCGACAA TACAATGGG**T CTGCCTACTG CCTCGGA 3' (bold being the target and complementary sequences for mouse PiT1). The authenticity of the LMP-derived vector was confirmed by sequence analysis. His-tagged FeLV-B RBD was produced from a pCDNA3 vector encoding the first 212 N-terminal amino acids of the SU of FeLV-B (G/A) fused to a C-terminal His-tag via a short linker (Dreyer, K. and Pedersen, L., unpublished work). C-terminally His-tagged A-MLV RBD encoded by a FBASALF-derived vector has been described elsewhere [31].

### Cells

The pre-osteoblastic murine cell line, MC3T3-E1 [22] (a kind gift from Dr. H. Kodama) and derivatives of this were cultured in Minimum Essential Medium alpha (MEMα) (Gibco BRL) supplemented with 10% FBS (Gibco BRL) and 1% Penicillin and Streptomycin (PS) (Gibco BRL) (MEMα-FBS-PS). Only MC3T3-E1 cells and derivatives of this cell line below the 5^th ^passage were used for experiments. The murine fibroblastic cell line, NIH3T3 (ATCC CRL-1658), and derivatives of this were cultivated in Dulbecco's Modified Eagle's Medium (DMEM) (Gibco BRL) supplemented with 10% Newborn Calf Serum (NCS) (Gibco BRL) and 1% PS (DMEM-NCS-PS). The 293T-derived retroviral packaging cell lines, Platinium Eco (PlatE) [32] and Phoenix™Eco (Orbigen) (http://www.stanford.edu/group/nolan/retroviral_systems/phx.html), and derivatives of the latter were cultivated in DMEM-FBS-PS. A-MLV (4070A isolate) and GALV (SEATO) pseudotypes of the β-galactosidase-encoding transfer vector G1BgSvN (GBN) [33] were obtained from the producer cell lines, PA317-GBN [29,34] and PG13-GBN [35], respectively. Both producer cell lines were cultivated in DMEM-NCS-PS. Vectors were harvested as supernatants from confluent producer cells and vector containing supernatants were filtered (0.45-μm pore size) and stored at −80°C until use. Dog osteosarcoma D17 (ATCC CCL-183) cells were cultivated in MEMα-FBS-PS. All plastic ware used for cell culture were from NUNC.

### PiT1-knock-down in MC3T3-E1 cells

Phoenix™-Eco cells were seeded at 1 × 10^4 ^cells/cm^2 ^in 100-mm-diameter dishes and transfected with LMP or LMP-MmPiT1shRNAmiR transfer vectors using the calcium-phosphate DNA precipitation method [36]. Two days after transfection, each 100-mm-diameter dish were transferred to two T175 flasks and selected with 1.25 μg/mL puromycin (Sigma-Aldrich) the first three days, and thereafter cultivated in 0.5 μg/mL puromycin. The Phoenix™-Eco-LMP and - LMP-MmPiT1shRNAmiR cells were grown to confluence, media were changed to MEMα-FBS-PS, and retroviral vector containing supernatants were harvested, filtrated (0.45-μm pore size), and stored at −80°C until use. MC3T3-E1 cells were seeded at 2.5 × 10^3 ^cells/cm^2^, and vector-containing supernatants supplemented with 2 μg/mL Polybrene (PB, Sigma-Aldrich) were added 3 times to the cells over 2 days. The cells were transferred to T175 flasks and selected with 4 μg/mL puromycin for 2 days before used in experiments.

### Establishment of LPiT1SN, LPiT2SN, and LXSN carrying MC3T3-E1 and NIH3T3 cells

Retroviral transfer vectors for transduction of MC3T3-E1 and NIH3T3 cells were produced in PlatE cells. PlatE cells were seeded at 4 × 10^6 ^cells/dish in 100-mm-diameter dishes in standard growth medium. The next day, the media were changed to fresh media and the cells were transfected with 30 μg of pLXSN, pLPiT1SN, or pLPiT2SN using the calcium-phosphate DNA precipitation method. The day after transfection, the media were changed to harvest media, and 24 hours later, vector containing supernatants were harvested, filtrated (0.45-μm pore size), and either used directly or stored at −80°C until use. A second harvest was made after an additional 24 hours using the same procedure. MC3T3-E1 cell populations carrying LPiT1SN, LPiT2SN, or empty vector LXSN were established and referred to as MC3T3-E1-LPiT1SN, MC3T3-E1-LPiT2SN, and MC3T3-E1-LXSN, respectively. NIH3T3 cell populations carrying LPiT1SN or empty vector LXSN were established and referred to as NIH3T3-LPiT1SN or NIH3T3-LXSN, respectively. The establishment of these cells was done as follows. MC3T3-E1 and NIH3T3 cells seeded at 6 × 10^3 ^cells/cm^2 ^in 60-mm-diameter dishes in standard growth media were transduced over a period of approximately 30 hours. Approximately two hours after seeding, media were changed to 3 mL per dish of filtered (0.45 μm) retroviral vector containing supernatants from Plat-E cells with 2 μg/mL PB. Additional transductions were carried out twice the following day. The transduced cells were selected with 400 μg active G418 per mL (Gibco BRL). The day after the last transduction, each 60-mm-dish was passaged into two T175 flasks in standard growth medium, and 48 hours later, 400 μg/mL active G418 was added, and G418 resistant cell populations established. The selected MC3T3-E1 cell populations were frozen at −80°C for at least 24 hours before their use in experiments.

### Proliferation assay

Cells were seeded in 4-well plates in quadruplicates in standard growth medium. At indicated days, the cells were detached from the wells using Trypsin-EDTA (5 × T) diluted in PBS from a stock of 10× Trypsin-EDTA (0.5% Trypsin, Gibco BRL), and re-suspended in 500 μL standard growth medium. Aliquots were mixed 1:1 with Trypan Blue Stain (Gibco BRL) and counted using a cell counting chamber.

### Cell cycle analysis

Cells were seeded in T25-flasks at 20,000 cells/cm^2^. After 24 hours, the cells were detached using 5 × T, pelleted by centrifugation, re-suspended in PBS, cooled on ice, mixed 1:1 with ice-cold 99.9% ethanol, incubated at least 10 minutes on ice and used for staining or stored at 4°C until staining. Staining was carried out by pelleting the cells by centrifugation, re-suspending them in 1 mL PBS containing 20 μg/mL RNase A followed by addition of 100 μL propidium iodide (1 mg/mL). The cells were then analyzed by flow cytometry (FL2 channel) using a FACSCalibur flow cytometer (Becton Dickinson). CellQuest software was used for acquisition. FlowJo software was used for subtraction of aggregates and doublets [37] and for data analysis employing the Watson-Pragmatic model.

### RBD binding assay

His-tagged RBDs were produced in 293T cells. Shortly, 293T cells were seeded at 4 × 10^6 ^cells/dish in 100-mm-diameter dishes and transfected the following day with 15 μg His-tagged FeLV-B RBD or His-tagged A-MLV RBD encoding expression plasmids together with 15 μg carrier plasmid (pUC18) per dish using the calcium-phosphate DNA precipitation method. At days 2 and 3 after transfection, RBD containing supernatants were harvested in standard growth medium, filtrated (0.45-μm pore size), and stored in aliquots at −80°C until use. For analysis of RBD binding, cells were detached using 1 mM EDTA in PBS and counted. Aliquots of 10^6 ^cells/sample were pelleted by centrifugation for 5 minutes at 1200 rpm. The cell pellets were re-suspended in 1 mL RBD-containing supernatant or standard growth medium, as control, and incubated 20 minutes at 37°C. The cells were then washed 2× in 5% FBS in PBS and incubated on ice with primary antibody, Mouse-Anti-Penta-His (Qiagen), diluted 1:400 in 50 μL 5% FBS in PBS/sample for 45 minutes. Then the cells were washed twice in ice-cold 5% FBS in PBS and incubated for at least 45 minutes in the dark with 1 μg/sample of PE-conjugated goat anti-mouse Ig (BD Pharma) diluted in 300 μL 5% FBS in PBS. Finally, the cells were washed 1× in ice cold 5% FBS in PBS, fixed in 1% paraformaldehyde (Ampliqon) for 10 minutes at RT, washed 1× in PBS, and re-suspended in 600 - 1000 μL PBS. The samples were analyzed using the FL2-H channel on a FACSCalibur flow cytometer. For data analysis, CellquestPro software was used.

### Transduction assay

MC3T3-E1-LXSN, -LPiT1SN, -LPiT2SN, NIH3T3-LXSN, -LPiT1SN, and D17 cells were seeded in 24-well plates with a density of 10^4 ^cells/cm^2 ^in standard growth media and analyzed for their susceptibility to transduction with GALV and A-MLV vector pseudotypes as previously described for titer determination using dilution series of vector-containing supernatants from PG13-GBN and PA317-GBN producer cells, respectively [11]. The transduction efficiency was evaluated as blue (β-galactosidase-positive) colony forming units (CFUs).

### qRT-PCR

Cells were lyzed on the plates and total RNA purified using RNAqueous^®^-4PCR kit (Applied Biosystems). The RNA samples were DNase1 treated and after inactivation of the DNase used directly for cDNA synthesis using the High Capacity cDNA Reverse Transcription Kit (Applied Biosystems) as recommended by the supplier. The samples were either stored at −20°C until use or used directly for qRT-PCR analysis. For qRT-PCR analyses, the following TaqMan^® ^Gene Expression Assays (Applied Biosystems) were used according to the protocol: Mouse PiT1 (Mm00489378_m1), mouse PiT2 (Mm00660197_m1), human PiT1 (Hs00965596_m1), human PiT2 (Hs01108472_m1) and, as endogenous control, mouse β-2-microglobulin (B2M)-VIC (Mm00437762_m1). The individual qRT-PCR reactions contained 10 μL TaqMan^® ^Fast Universal PCR Master Mix ((Applied Biosystems), 1 μL TaqMan^® ^Gene Expression Assay, 1 μL B2M, and 8 μL cDNA (approximately 10 ng). All samples were made in triplicates for all genes analyzed. The efficiencies of each set of primers were determined on dilution series of cDNA and were used in calculations of relative gene expression as described in ref. [38]. The PCR cycles employed were: 95°C for 10 minutes, 40 cycles of 95°C for 1 second, and 60°C for 20 seconds.

### ^32^P_i_-transport assay

Cells were seeded in 4-well plates, the next day, they were washed in P_i_-free DMEM (Gibco BRL) and hereafter incubated 5 minutes in 37°C warm serum-free, P_i_-free DMEM containing KH_2_^32^PO_4 _(900-1100 mCi/mmol, Perkin Elmer) with or without added non-radioactive P_i_. The endconcentrations of P_i _in the uptake experiments were 5 μM, 100 μM, or 300 μM P_i_. The cells were then washed 3× in ice cold PBS or serum-free, P_i_-free DMEM, and lyzed in 0.5% (w/v) SDS in PBS or in 0.5% (v/v) Triton X-100, and 2/3 of the lysate was mixed with 5 mL Ultima Gold Liquid Scintillation Counter Cocktail (Perkin Elmer) or Optiphase "Hisafe" 3 (Perkin Elmer) and measured in liquid scintillation counters. The differences in washing and lysis procedures did not influence the results (unpubl. observation). The rest of the sample was stored at −20°C until used for protein determination using the BCA™ protein assay kit (Pierce).

### Soft agar assay

NIH3T3-LXSN and NIH3T3-LPiT1SN cells were pre-incubated for two days in either standard growth medium or DMEM-FBS-PS. Base agar was made by melting 1% agar (SeaPlaque^®^GTG^®^Agarose, Lonza), cooling it to 40°C in a water bath and mixing it with an equal volume of 40°C warm 2× DMEM (Gibco BRL) supplemented with 3.7 g NaHCO_3 _(MERCK), 2% PS, and 20% FBS (2 × DMEM/FBS) or 20% NCS (2 × DMEM/NCS); 0.5 mL base agar was plated per well in 24-well plates and allowed to set at 4°C. NIH3T3-LXSN or NIH3T3-LPiT1SN cells grown in either DMEM-FBS-PS or DMEM-NCS-PS were loosened with 5 × T and diluted to a concentration of 10^5 ^cells/mL, and 50 μL of the cells were mixed with 1 mL melted 40°C 0.7% agar and 1 mL 40°C 2 × DMEM/FBS or 2 × DMEM/NCS, respectively, and 0.5 mL per well were seeded on top of the solidified base agar containing DMEM/FBS or DMEM/NCS, respectively. When the top agar had set, 0.25 mL DMEM-NCS-PS or DMEM-FBS-PS were added to each well. The dishes were incubated for 18 days at 37°C and 5% CO_2_. Colonies were stained by addition of 200 μL 0.005% Crystal Violet (MERCK) to each well followed by 1 hour incubation at room temperature before counting the colonies under a microscope.

### Statistical analysis

The hypothesis that two mean values were identical was tested by a two-tailed Student's *t*-test; a p-value <0.05 was considered statistically significant.

## Abbreviations

P_i_: inorganic phosphate; PiT: P_i _transporter; hPiT1: human PiT1; mPiT1: murine PiT1; hPiT2: human PiT2; mPiT2: murine PiT2; GALV: gibbon ape leukemia virus; FeLV-B: feline leukemia virus subgroup B; A-MLV: amphotropic murine leukemia virus; MEF: mouse embryonic fibroblast; CFU: colony forming unit; RBD: receptor binding domain; qRT-PCR: quantitative RT-PCR.

## Competing interests

The authors declare that they have no competing interests.

## Authors' contributions

KB: Participated in conceiving the study and its design, established NIH3T3-derived cells, performed the majority of the shown experiments and data analyses of these, and made the draft of the manuscript. NJ: Participated in conceiving part of the study design and, together with IBK, established MC3T3-E1-derived cells and made the initial characterization, and contributed to the manuscript draft. IBK: Participated in conceiving part of the study design and, together with NJ, established MC3T3-E1-derived cells and made the initial characterization, and contributed to the manuscript draft. MW: Performed PiT1 knock-down in MC3T3-E1-derived cells, and performed part of the characterization of the cells, and contributed to the manuscript draft. LEP: Participated in conceiving part of the study design, established the qRT-PCR and qRT-PCR data analyses protocols, and performed part of the data analyses, and contributed to the manuscript draft. CH: Participated in conceiving part of the study design, made the mPiT1 knock-down vector, and part of the P_i _uptake characterization studies, and contributed to the manuscript draft. LP: Participated in conceiving the study and its design, coordinated the work, and finalized the manuscript. All authors read and approved the final manuscript.
